# Cardiovascular history and risk of idiopathic Parkinson’s disease: a cross-sectional observational study

**DOI:** 10.1186/s12868-024-00875-y

**Published:** 2024-07-08

**Authors:** Shubhra Acharya, Andrew I. Lumley, Yvan Devaux, NCER-PD Consortium, Muhammad Ali, Nancy E. Ramia, Giuseppe Arena, Rudi Balling, Michele Bassis, Regina Becker, Ibrahim Boussaad, Piotr Gawron, Soumyabrata Ghosh, Enrico Glaab, Elisa Gómez De Lope, Valentin Groues, Anne Grünewald, Wei Gu, Michael Heneka, Sascha Herzinger, Jacek Jaroslaw Lebioda, Yohan Jaroz, Quentin Klopfenstein, Zied Landoulsi, Tainá M. Marques, Patricia Martins Conde, Patrick May, Francoise Meisch, Sarah Nickels, Marek Ostaszewski, Clarissa P. C. Gomes, Sinthuja Pachchek, Armin Rauschenberger, Rajesh Rawal, Dheeraj Reddy Bobbili, Kirsten Roomp, Isabel Rosety, Stefano Sapienza, Venkata Satagopam, Sabine Schmitz, Reinhard Schneider, Jens Schwamborn, Ekaterina Soboleva, Rebecca Ting Jiin Loo, Christophe Trefois, Carlos Vega, Maharshi Vyas, Paul Wilmes, Evi Wollscheid-Lengeling, Jochen Klucken, Rejko Krüger, Claire Pauly, Lukas Pavelka, Linda Hansen, Gilles van Cutsem, Geeta Acharya, Gloria Aguayo, Myriam Alexandre, Wim Ammerlann, Katy Beaumont, Camille Bellora, Jessica Calmes, Lorieza Castillo, Gessica Contesotto, Daniela Esteves, Guy Fagherazzi, Jean-Yves Ferrand, Marijus Giraitis, Jérôme Graas, Gaël Hammot, Anne-Marie Hanff, Estelle Henry, Michael Heymann, Alexander Hundt, Sonja Jónsdóttir, Pauline Lambert, Victoria Lorentz, Paula Cristina Lupu, Guilherme Marques, Deborah Mcintyre, Chouaib Mediouni, Myriam Menster, Maura Minelli, Ulf Nehrbass, Fozia Noor, Magali Perquin, Rosalina Ramos Lima, Eduardo Rosales, Estelle Sandt, Margaux Schmitt, Amir Sharify, Kate Sokolowska, Hermann Thien, Johanna Trouet, Olena Tsurkalenko, Michel Vaillant, Mesele Valenti, Guy Berchem, Nico Diederich, Liliana Vilas Boas, Gelani Zelimkhanov, Laura Longhino, Romain Nati, Beatrice Nicolai, Elodie Thiry, Friedrich Mühlschlegel, Alexandre Bisdorff, Rene Dondelinger, Sylvia Herbrink, Roseline Lentz, Michele Hu, Richard Wade-Martins, Clare Mackay, Daniela Berg, Kathrin Brockmann, Thomas Gasser, Inga Liepelt, Brit Mollenhauer, Katrin Marcus, Robert Liszka, Walter Maetzler, Mariella Graziano, Nadine Jacoby, Jean-Paul Nicolay, Laure Pauly, Michel Mittelbronn

**Affiliations:** 1 Cardiovascular Research Unit, Department of Precision Health, Luxembourg Institute of Health, 1445 Strassen, Luxembourg; 2 Faculty of Science, Technology and Medicine, University of Luxembourg, 4365 Esch-Sur-Alzette, Luxembourg

**Keywords:** Parkinson’s disease, Cardiovascular health, Comorbidities, Sex-differences

## Abstract

**Background:**

Parkinson's disease (PD), while often associated with its distinctive motor symptoms, can also exert a notable impact on the cardiovascular system due to the development of severe autonomic dysfunction. One of the initial indicators of PD is the appearance of cardiovascular dysautonomia. As such, it is vital to monitor and manage cardiovascular health of individuals with PD, as it may have clinical implications in the development of commonly recognized motor and non-motor aspects of the disease. To study the association of history of cardiovascular disease (CVD) with occurrence and severity of PD, here, we lend data on the association of CVD history with the frequency and the occurrence of idiopathic PD (iPD) using data from the Luxembourg Parkinson’s study (iPD n = 676 patients and non-PD n = 874 controls).

**Results:**

We report that patients with a history of CVD are at high risk of developing iPD (odds ratio; OR = 1.56, 95% confidence interval; CI 1.09–2.08). This risk is stronger in males and remains significant after adjustment with confounders (OR 1.55, 95% CI 1.05–2.30). This increased susceptibility to iPD is linked to the severity of iPD symptoms mainly the non-motor symptoms of daily living (MDS-UPDRS I) and motor complications (MDS-UPDRS IV) in the affected individuals.

**Conclusion:**

Individuals with history of CVD have a high risk of developing severe forms of iPD. This observation suggests that careful monitoring and management of patients with a history of cardiac problems may reduce the burden of iPD.

## Background

With an increasing life expectancy, there is an increased prevalence of age-related disease burden. Among the diseases associated with age, neurodegenerative diseases such as Parkinson’s disease (PD) and cardiovascular diseases (CVD) remain major causes of death worldwide. The interplay between the nervous system and the cardiovascular system has been discussed for years [1] and recent papers rejuvenate the neurocardiology field [2–4]. PD is the second most common multifactorial age-related disorder that arises due to progressive degeneration of dopaminergic neurons in the substantia nigra pars compacta in the midbrain. Dopaminergic neurons are responsible for the secretion of neurotransmitter dopamine, responsible for maintenance of motor activities. The loss of dopamine in the midbrain leads to impaired motor skills and appearance of motor symptoms in PD patients. This neuronal degeneration could be governed by several interlinked biological processes, such as protein aggregation, genetic mutations or mitochondrial dysfunction [5–7]. Mutations in genes like LRRK2 and SNCA are directly associated with increased PD risk by altering protein function [8]. Additionally, epigenetic mechanisms like DNA methylation have been shown to influence gene expression, potentially affecting dopamine production and PD progression [9]. The combination of genetic and epigenetic mechanisms contributes to an individual’s susceptibility to developing PD. As the disease progresses, other parts of the brain might also be affected leading to the development of non-motor symptoms. Due to the presence of several non-motor symptoms in early stages of PD, whether PD starts in the body first or the brain is still a matter of debate [1]. Our research aims to explore the body-first PD hypothesis highlighting the need to examine the brain and heart axis in the context of idiopathic PD (iPD) [2]. As part of the natural process of neurodegeneration, older individuals often exhibit symptoms resembling those of PD, such as tremors and bradykinesia, even though they might not receive a PD diagnosis [10]. These symptoms may arise from underlying vascular conditions such as cardiovascular abnormalities. Therefore, it becomes crucial to closely monitor these individuals, as cardiovascular issues may potentially contribute to the progression of PD [11]. Additionally, the functional and molecular links between PD and cardiovascular conditions needs to be thoroughly examined [2]. This association could involve several multifaceted mechanisms involving autonomic dysfunction and shared risk factors like aging, inflammation, comorbidities, medications, physical activity or genetic predispositions. Cardiovascular dysautonomia which includes orthostatic hypotension, hypertension and heart rate variability is observed as one of the early signs of PD [12]. Other cardiac complications in PD include cardiomyopathy, coronary heart disease, arrhythmias, sudden cardiac death and Levodopa-induced CVD [13, 14]. These cardiac defects additionally act as risk factors in developing sudden unexpected death in PD patients. In an American cross –sectional study, Zesiewicy et al. observed that elderly patients with PD experienced heart failure twice as often as those without PD [15]. Likewise, a Korean population-based study has shown that PD was associated with high risk of developing CVD [16]. Contrarily, an inverse relation between CVD and PD was established in a Danish cohort, where patients with myocardial infarction had 20 and 28% decreased risk of developing PD and secondary Parkinsonism, respectively [17]. These studies shed light on the need for diverse population based research on the relationship between CVD and PD [18]. Given the potential impact of cardiovascular factors on PD progression, investigating this association is crucial.

The Luxembourg Parkinson’s (LuxPARK) study, that provides a comprehensive coverage of PD and other parkinsonian syndromes within Luxembourg and the Greater Region [19], offers a unique opportunity to explore the relationship between CVD history and the onset and severity iPD. Thus, we hypothesize that a history of CVD is associated with both the occurrence and severity of iPD. In the present study, we aim to examine the frequency of CVD in patients with iPD and assess relationship between the history of CVD and the onset and severity of iPD in the Luxembourg Parkinson’s (LuxPARK) study. Here, we consider both cardiac and cerebral health when evaluating iPD, as well as the potential sex-specific differences in iPD susceptibility.

## Methods

### Study design

This cross-sectional observational study performed using the clinical data from the LuxPARK study is reported following the STROBE guidelines [20]. The data in LuxPARK study were collected between 2015 and 2020. The protocol was performed according to the principles of the Declaration of Helsinki. All participants signed an informed consent and the study was approved by the national ethics committee (CNER Ref: 201407/13 and 20140713-SU3) and Data Protection Committee (CNPD Ref: 446/2017) [19]. The data was anonymized.

### Study participants

Participants from the LuxPARK study were divided into iPD (n = 676) or non-PD (n = 874) controls. For iPD classification and inclusion in the LuxPARK study, the patients met the United Kingdom Parkinson’s Disease Society Brain Bank Clinical Diagnostic Criteria [21]. The patients who did not meet the above criteria were classified as atypical PD and were not included in the present study. Non-PD controls were participants recruited based on the exclusion criteria, such as, presence of a neurodegenerative disorder, active cancer, being under the age of 18, or being pregnant. An independent sample size calculation was performed for the LuxPARK study for baseline comparisons to allow finding significant differences between the two groups [19]. Briefly, sample size calculations were based on estimated PD prevalence and incidence in Luxembourg which suggested a sample size of 800 individuals per group to be sufficient to detect a moderate difference in prevalence between groups using a chi-square test with 80% power and a 5% two-sided significance level [19].

The number of iPD and non-PD controls recruited as a part of LuxPARK study during the study period determined the sample size for the present study. Baseline clinical visits (visit 1) for all participants (iPD and non-PD) took place between 2015 and 2020.

### Clinical assessment and data collection

Clinical data of study participants were obtained at baseline. The clinical assessment was conducted via a health questionnaire performed by an experienced movement disorders specialist. This included demographic details such as age, sex, height, weight, and smoking history. Medical history included presence or absence of comorbidities at the time of baseline visit or in the past. For this study, the comorbidities studied were history of diabetes, CVD and hypertension. Further, detailed neurological examination for disease severity was performed using the Movement Disorder Society- Unified Parkinson’s Disease Rating Scale (MDS-UPDRS; [22]). Evaluation of general cognitive symptoms was achieved by Montreal Cognitive Assessment (MoCA; [23]). The effect on the autonomic symptoms was assessed using the Scales for Outcomes in Parkinson’s disease (SCOPA-AUT; [24]).

### Statistical analysis

Statistical analyses were performed using R version 4.0.3. The missForest algorithm, utilizing random forests, was applied to impute missing values in both continuous and categorical variables. Following imputation, summary statistics were reviewed to confirm consistency with the original data distribution, and imputed values were subsequently assigned to their respective variables for further analysis. Continuous variables were rank normalized using ‘RankNorm’. Pearson’s Chi-square test was used to evaluate differences in frequencies between non-PD and iPD groups. R packages Hmisc, rms, lmtest and glmtoolbox were used to perform logistic regression analysis. The primary outcome was the un-adjusted (univariate) and adjusted (multivariate) relationship between CVD history and iPD occurrence. Common disease-associated risk factors such as age, sex, body mass index (BMI), history of diabetes, history of hypertension and smoking history were used as confounders for adjustment in the multivariate logistic regression analysis. The association between iPD, CVD history and autonomic symptoms was studied using the analysis of the SCOPA-AUT score. The score was log2 transformed and the difference between the groups was analyzed by Mann–Whitney Rank Sum test. The secondary outcome was the association of CVD history with iPD severity as determined by MDS-UPDRS scores and MoCA. Disease duration was also added as a confounder in the disease severity analysis. Lastly, for all of the analysis sex specific association was analyzed by dichotomizing the patients based on sex.

## Results

### Clinical characteristics of the participants

1550 participants were included in the present study that included 874 non-PD controls and 676 iPD patients. The clinical characteristics of the participants in LuxPARK cohort showed that iPD patients have a severe clinical profile compared to non-PD controls. They were older (65.97 ± 10.39 years in iPD vs 59.21 ± 12.43 years in non-PD; p < 0.001), predominantly male (65% in iPD vs 51% in non-PD) and showed a higher proportion of CVD history (18.9% in iPD vs 9.3% in non-PD), hypertension (42.1% in iPD vs 34.3% in non-PD) and diabetes (9% in iPD vs 6.1% in non-PD). Patients with iPD as expected, displayed severe motor (MDS-UPDRS) and autonomic symptoms (SCOPA-AUT) and lower score on the MoCA, indicating poor cognitive function. Further, there was no significant difference between the smoking prevalence (p = 0.08) and the BMI between the two groups (p = 0.4). The proportions of non-PD and iPD patients with CVD history and co-morbidities are described in Table 1. Concerning the sex distribution and CVD history, within the iPD group, 128 patients had history of CVD, of which 102 (80%) were males and 26 (20%) were females. Meanwhile, within the non-PD controls 82 individuals had history of CVD, of which 29 (35%) were females and 53 (64%) were males, showing a higher incidence of CVD in men compared to women in both non-PD and iPD groups. Lastly, non-PD controls had significant other comorbidities such hypertension (n = 300; 60% males) and diabetes (n = 54; 64% males).

**Table 1:** Demographic and clinical characteristics of the study population

	Non-PD controls	iPD	*p* value
n	874	676	
Age	59.21 ± 12.43	65.97 ± 10.39	< 0.001
Males n (%)	451 (51%)	444 (65%)	< 0.001
CVD n (%)	82 (9.3%)	128 (18.9%)	< 0.001
Hypertension n (%)	300 (34.3%)	285 (42.1%)	0.002
Diabetes n (%)	54 (6.1%)	67 (9%)	0.007
Smokers n (%)	420 (48%)	295 (43.6%)	0.08
BMI	27.3 ± 5.25	27.53 ± 4.70	0.4
MDS-UPDRS I	4.9 ± 4.6	9.8 ± 6.2	< 0.001
MDS-UPDRS II	1.3 ± 2.4	10.2 ± 7.1	< 0.001
MDS-UPDRS III	3.6 ± 3.6	32.5 ± 14.7	< 0.001
MDS-UPDRS IV	0.0007 ± 0.01	1.5 ± 3.5	< 0.001
SCOPA-AUT	7.9 ± 6.1	14.3 ± 7.9	< 0.001
MoCA	27.05 ± 2.56	25.3 ± 3.2	< 0.001

Numbers are represented as mean ± SD

*BMI* Body Mass Index, *iPD* idiopathic Parkinson’s disease, *MDS-UPDRS* Movement Disorder Society- Unified Parkinson’s Disease Rating Scale, *SCOPA-AUT* Scales for Outcomes in Parkinson’s disease, *MoCA* Montreal Cognitive Assessment Test

### Association of CVD history with the occurrence of iPD

**Fig. 1:**
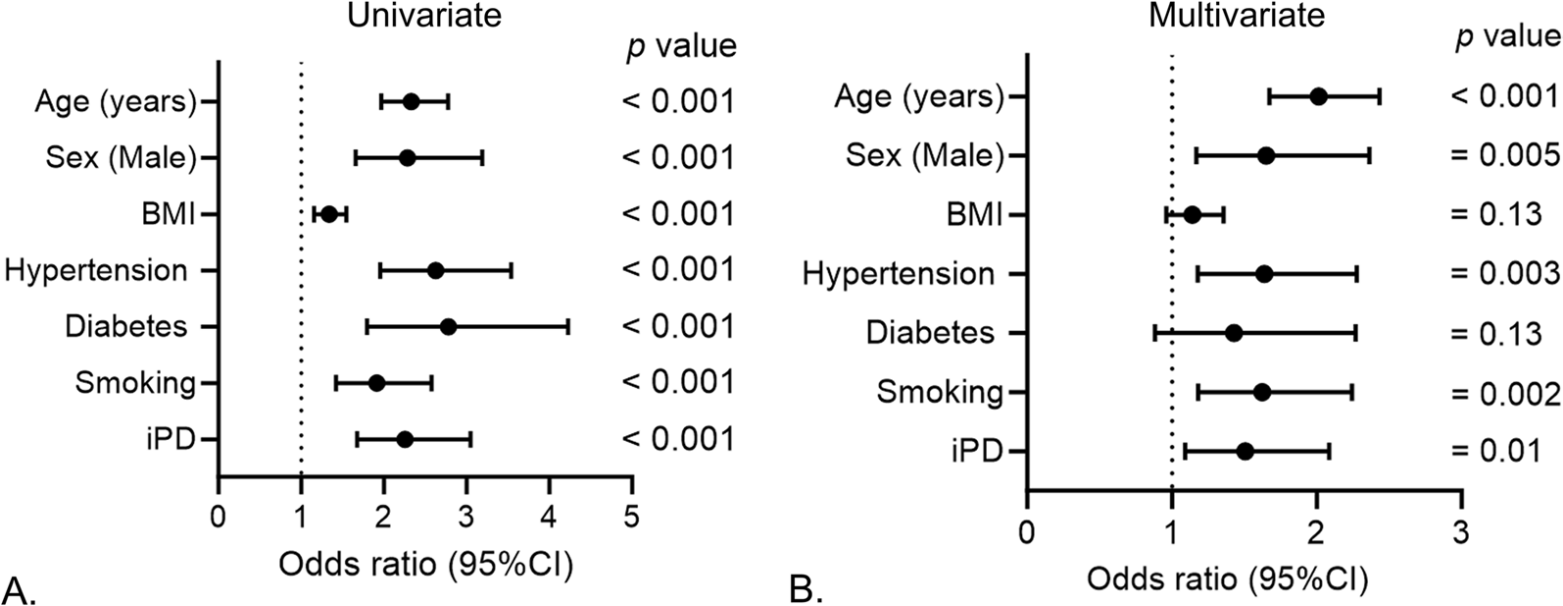
Association of CVD history with the occurrence of iPD. **A** Univariate and **B** multivariate logistic regression to study the association between history of CVD and occurrence of iPD. Age, sex, BMI, hypertension, diabetes and smoking were included as confounders in the multivariate models

**Fig. 2:**
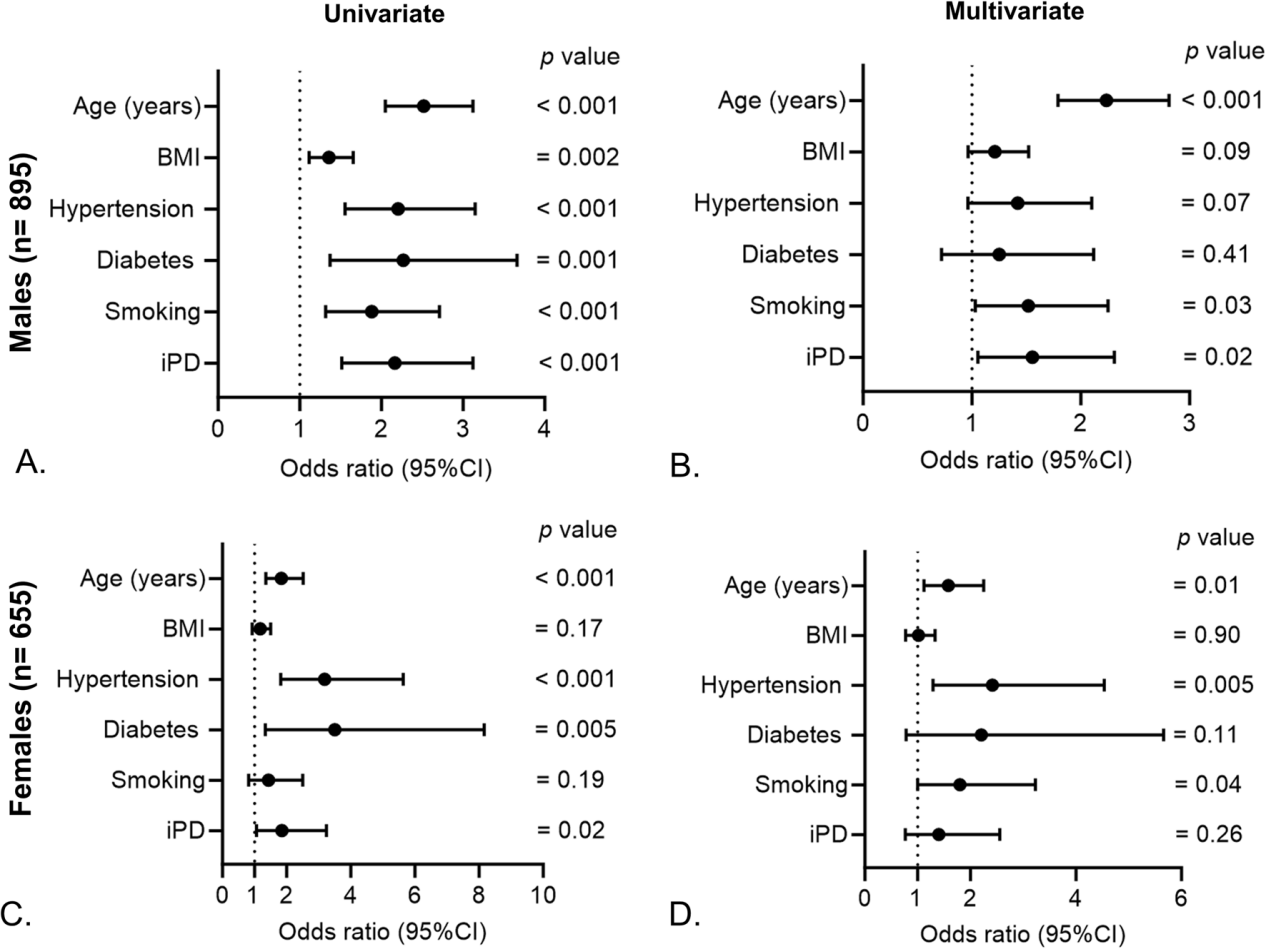
Sex-specific association of CVD history with the occurrence of iPD. **A** Univariate and **B** multivariate logistic regression to study the association between history of CVD and occurrence of iPD in males. **C** Univariate and **D** multivariate logistic regression to study the association between history of CVD and occurrence of iPD in females. Age, BMI, hypertension, diabetes and smoking were included as confounders in the multivariate models

**Fig. 3:**
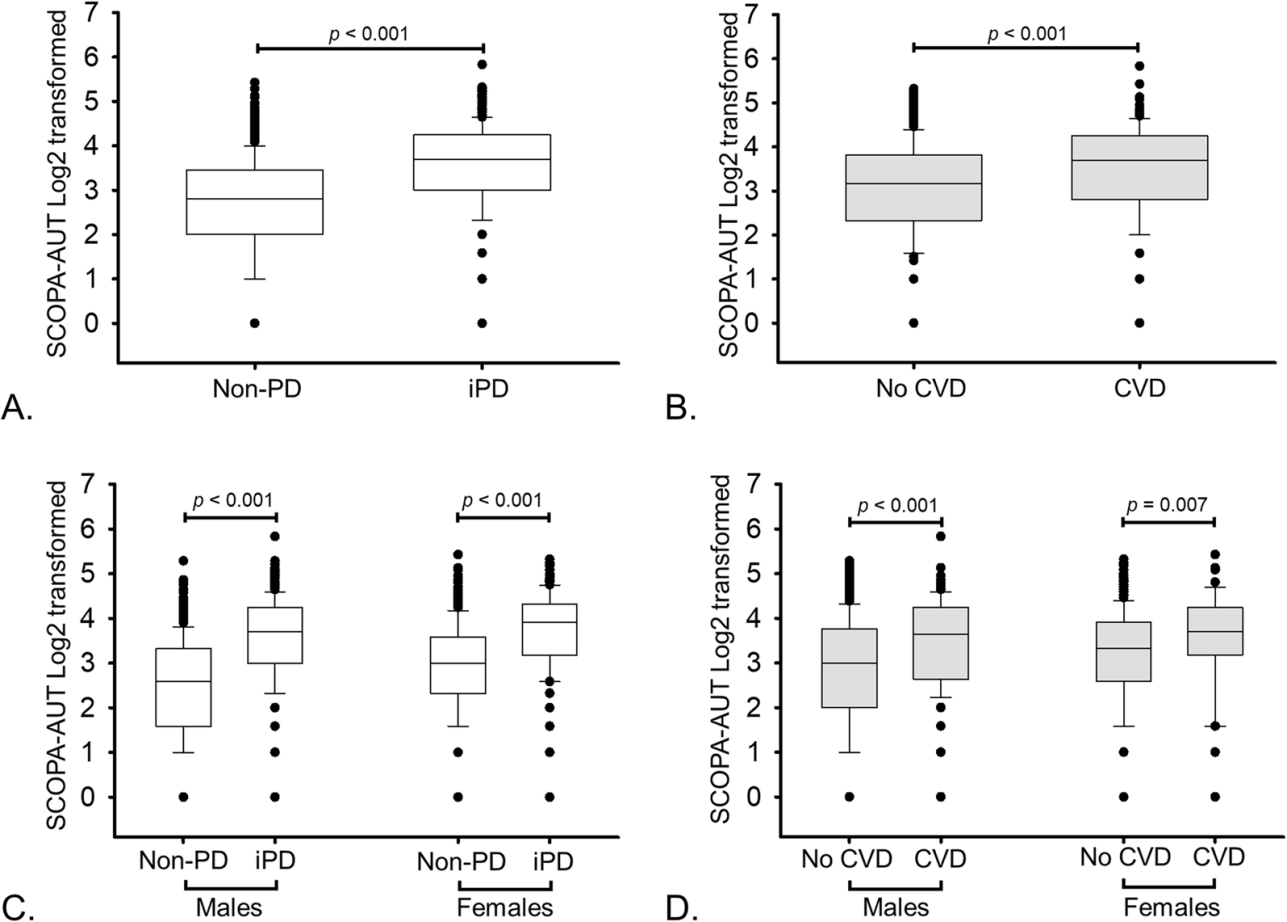
Autonomic symptoms as assessed by the SCOPA-AUT score in (**A**) non-PD controls (n = 874) compared to iPD patients (n = 676), **B** participants who did not have history of CVD (n = 1337) compared to who had history of CVD (n = 213). **C** Sex specific association of SCOPA AUT with iPD **D** Sex specific association of SCOPA AUT with history of CVD. Statistical analysis was performed using the Mann–Whitney Rank Sum test

To study the association of CVD history with the occurrence of iPD we performed univariate and multivariate logistic regression analysis. In univariate logistic regression analysis, participants that had a history of CVD had a higher risk of iPD occurrence (odds ratio OR = 2.25, 95% confidence interval (CI) = 1.67–3.04, p-value < 0.001; Fig. 1A). This association remained significant after adjusting for the common disease associated confounding factors such as age, BMI, hypertension, diabetes and smoking (OR = 1.56, 95% CI 1.09–2.08, p = 0.013; Fig. 1B). As the overall incidence of CVD history in the cohort (non-PD + iPD) was higher in men (n = 155 out of 895; 17.3%) than in women (n = 55 out of 655; 8.3%; p < 0.001), we investigated the sex-specific association of CVD history with iPD occurrence. In the univariate analysis, CVD history was associated with iPD occurrence in both males and females (Fig. 2A and C). However, this association remained significant only in males after adjustment with confounders (OR = 1.55, 95% CI 1.05–2.30, p = 0.026; Fig. 2B and D). As autonomic dysfunction is a common risk factor for both iPD and CVD, we studied its association with both the diseases in the cohort and observed strong association between iPD (Fig. 3A), CVD (Fig. 3B), and autonomic symptoms at baseline in both males and females (Fig. 3C and D).

### Association of CVD history with iPD severity

**Fig. 4:**
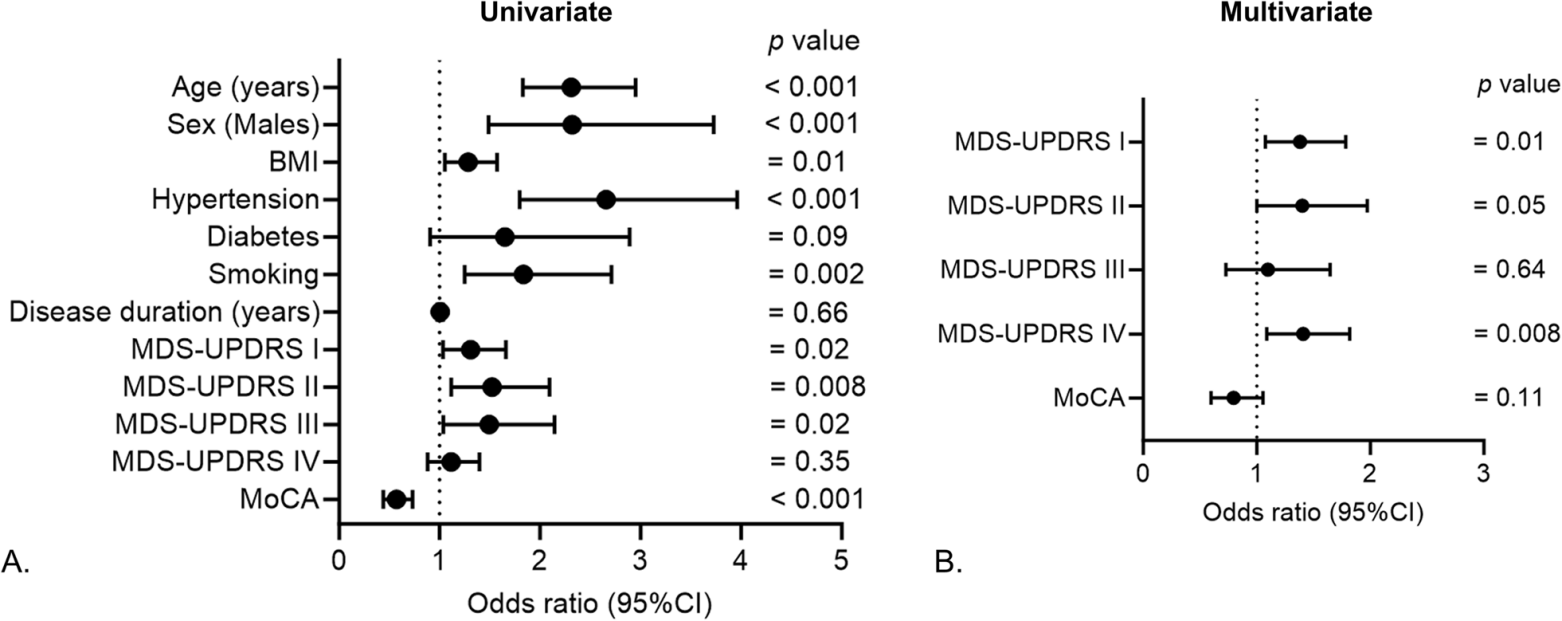
Association of CVD history with iPD severity. **A** Univariate and **B** multivariate logistic regression to study the association between history of CVD and disease severity scores in iPD patients. Age, sex, BMI, hypertension, diabetes, smoking and disease duration were included as confounders in the multivariate models

**Fig. 5:**
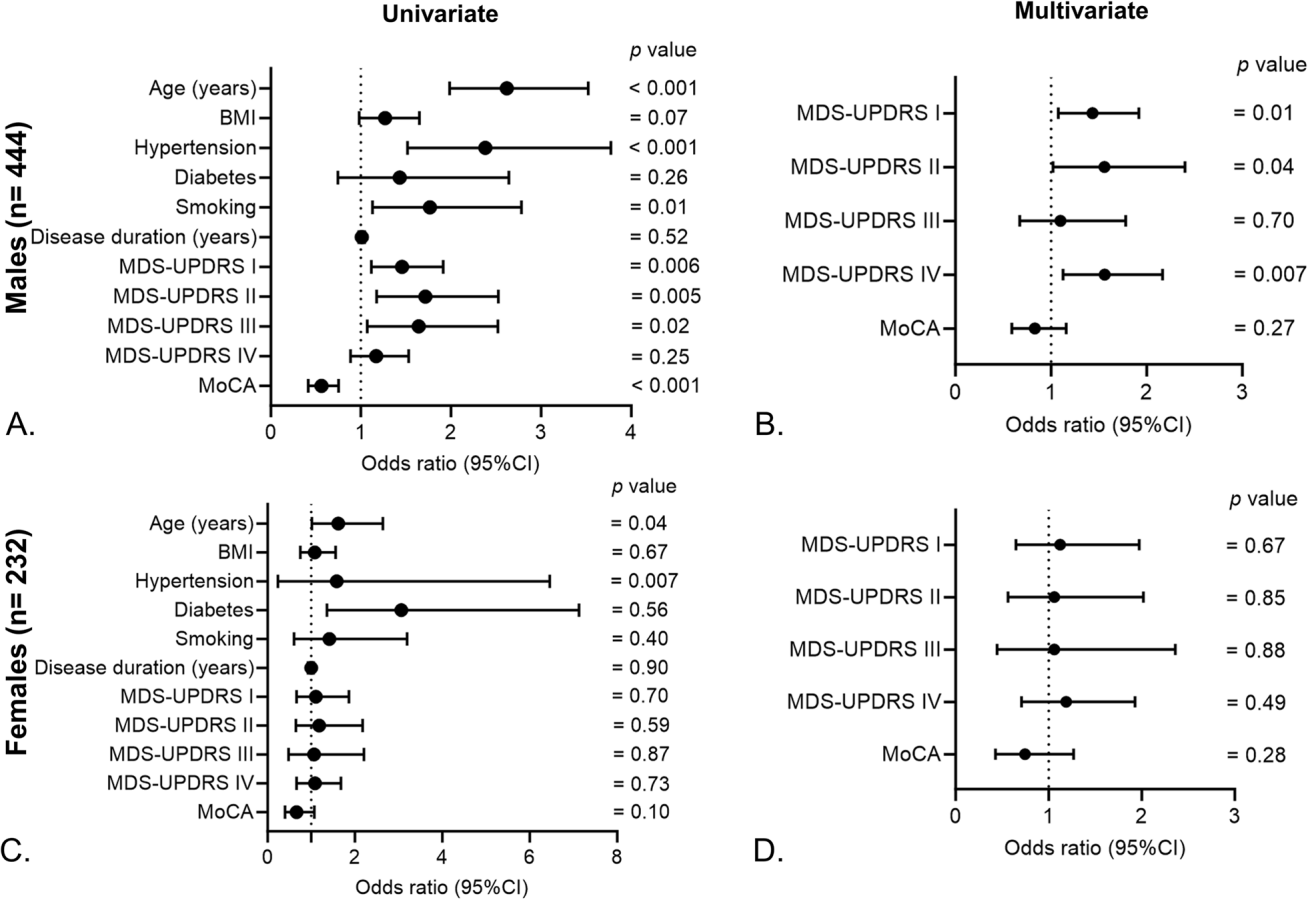
Sex-specific association of CVD history with iPD severity. **A** Univariate and **B** multivariate logistic regression to study the association between history of CVD and disease severity scores in male iPD patients. **C** Univariate and **D** multivariate logistic regression model to study the association between history of CVD and disease severity scores in female iPD patients. Age, BMI, hypertension, diabetes, smoking and disease duration were included as confounders in the multivariate models

In an effort to gain a deeper understanding of how a patient's cardiac history relates to the severity of their iPD symptoms, we evaluated the extent of symptom severity using distinct assessment tools such as MDS-UPDRS scores and MoCA. In univariate logistic regression analysis, history of CVD in iPD patients was associated with MDS-UPDRS I (OR = 1.30, 95% CI 1.03–1.65, p = 0.02), MDS-UPDRS II (OR = 1.52, 95% CI 1.11–2.09, p = 0.008), MDS-UPDRS III (OR = 1.49, 95% CI 1.03–2.14, p = 0.02 and MoCA (OR = 0.57, 95% CI 0.44–0.73, p < 0.001) but not with MDS-UPDRS IV (OR = 1.11, 95% CI 0.88–1.39, p = 0.35; Fig. 4A). Upon adjustment with confounders, MDS-UPDRS I (OR = 1.38, 95% CI 1.07–1.78, p = 0.01) and MDS-UPDRS IV (OR = 1.43, 95% CI 1.09–1.86, p = 0.008) remained significantly associated with CVD history in iPD patients (Fig. 4B). Upon dichotomizing iPD patients into two groups based on sex, CVD history was significantly associated with disease severity scores (MDS-UPDRS I, II and IV) in males (Fig. 5A, B), with no such correlation observed in females (Fig. 5C, D).

## Discussion

Our findings from the LuxPARK cohort support the established clinical profile of iPD patients compared to non-PD controls. As expected, the iPD group displayed a male predominance, aligning with well-documented characteristics of the disease. This sex disparity in PD patients could be attributed to hormonal differences, as estrogen, a female sex hormone, has been shown to possess neuroprotective qualities [25]. Additionally, high exposure to occupational toxins and X chromosome linked genetic factors could contribute to more prevalence in males [26]. Further, in our study, we observed that patients with iPD have a higher frequency of underlying CVD and cardiovascular co-morbidities such as hypertension and diabetes, compared to non-PD controls, supporting the existence of functional links between cardiovascular, cerebrovascular and neurological health in iPD patients. Furthermore, using logistic regression models we show a significant association between the history of cardiac problems and the occurrence of iPD. With males being at a greater risk for CVD [27], our investigation further reveals that males with CVD history are exposed to a greater risk of iPD development than females with CVD history. The autonomic nervous system is mainly involved in controlling the heart rate. In the present study, we observed increased autonomic dysfunction in patients with iPD compared to the non-PD controls. Finally, to understand how prior cardiac defects could influence iPD severity, we further studied its association with CVD history. Our analysis of disease severity scores in iPD patients revealed a significant association of CVD history with MDS-UPDRS I and MDS- UPDRS IV (Unified Parkinson's Disease Rating Scale Part IV) scores.

While the primary pathogenesis of PD has long been associated with neurodegenerative processes, emerging evidence suggests that vascular factors play a significant role in the development and progression of PD [11]. Our study supports the body first hypothesis of PD development and shows that a history of CVD might be a risk factor for development of severe forms of PD. Similar to our findings, in a previous study, patients with iPD had frequent autonomic dysfunction, with an involvement of parasympathetic nervous system and frequent involvement of sympathetic cardiovascular dysfunction [28]. Our results suggest that underlying cardiovascular problems could be a risk factor for developing iPD, especially in males. This could be attributed to common disease-associated risk factors between CVD and PD, chronic inflammation because of the underlying cardiac problems or due to CVD induced vascular damage leading to reduced blood flow to the brain causing the neuronal death [29, 30]. Therefore, CVD history as a risk and sex-specific differences must be taken into account when assessing patients with suspected iPD and developing preventive measures.

Understanding the progression of iPD can provide valuable insights into the management and care of patients. Our findings suggest that the presence of CVD is linked to early non-motor symptoms (MDS-UPDRS I) and secondary motor complications (MDS-UPDRS IV) in iPD patients. These results align with emerging evidence that non-motor symptoms, including cardiac autonomic dysfunction, observed in early stages of PD, can significantly impact overall clinical representation [31]. Early signs of autonomic dysfunction, like constipation or urinary problems, could precede motor symptoms, potentially indicating involvement of the autonomic nervous system early in the disease process. Additionally, problems with blood pressure regulation causing dizziness could hinder mobility, potentially accelerating disease progression [32]. As such, identifying and managing these non-motor symptoms, particularly in iPD patients with CVD, becomes crucial for comprehensive care. Additionally, the secondary motor complications such as dystonia and dyskinesia may be induced due to several reasons, including drug-induced side effects (for example, levodopa exposure) [33]. Further, levodopa has been shown to initiate additional adverse effects, notably, cardiovascular defects [13]. Thus, the association between CVD with MDS-UPDRS IV could potentially indicate the deleterious effects of levodopa, leading to aggravation of cardiovascular symptoms in these patients, thus causing the development of drug-induced motor complications. Therefore, vigilant monitoring of individuals with pre-existing CVD who are receiving levodopa or similar medications for iPD is necessary. Next, our observation that MDS-UPDRS I, MDS-UPDRS II and MDS-UPDRS IV scores were significantly associated with CVD history in males highlights the sex-specific effects associated with iPD and CVD. While the absence of statistical significance in the female iPD patients might be in part due to low number of females in the study cohort, the gender-specific association suggests that CVD history may exert different influences on disease severity in males compared to females. Our results raise intriguing questions about the potential hormonal, genetic, or environmental factors that might underlie these disparities.

While our study provides valuable insights, it is important to note that there are limitations in our work. Firstly, the study was based on patient reported questionnaires, where the patients were asked for ‘History of Present Illness’ including ‘Cardiovascular disease’, ‘Hypertension’ or ‘None of the above’. Additionally, the duration of these pre-existing co-morbidities was not evaluated in the study. As such, it would be meaningful to perform a detailed assessment of the types of CVD to further study the implications of different types of cardiac defects in patients with iPD. Secondly, our study is observational in nature, and therefore, causality cannot be established. The relationship between CVD and iPD severity may be bidirectional, with PD potentially contributing to the development or aggravation of CVD. However, this possibility requires further prospective evaluation to delineate the cause and effect relationship between vascular health and neurodegenerative processes in PD.

Despite these limitations, our study further strengthens our knowledge regarding the association between CVD and iPD. We have demonstrated that the presence of CVD is significantly associated with iPD occurrence and with increased disease severity, particularly in terms of early non-motor symptoms and secondary motor complications in iPD patients. Moreover, we have highlighted sex-specific differences in this association, with males showing a significant link between CVD history and higher MDS-UPDRS I, MDS-UPDRS II, and MDS-UPDRS IV scores. Our findings highlight the importance of holistic patient care for individuals with iPD, taking into account both the neurological and cardiovascular aspects of their health. The shared risk factors between PD and CVD and the bidirectional relationship between the two conditions highlight the need of comprehensive management strategies targeting both neurological and cardiovascular health. To closely monitor patients, easy to perform tests like the Head-up tilt test to examine the heart rate variability could be measured during the clinical visits [34]. Additionally, at-home blood pressure diaries for patients before the clinical follow-up could help personalizing the treatment of these patients.

## Conclusions

Our study contributes insights into the complex relationship between iPD and CVD, paving the way for improved care and tailored treatment strategies for affected individuals. Further research is needed to elucidate the underlying mechanisms driving these associations and to explore potential interventions that can alleviate the impact of CVD on iPD progression, especially in sex-specific contexts.

## Data Availability

Patient data used in the preparation of this manuscript were obtained from the National Centre of Excellence in Research on Parkinson’s Disease (NCER-PD). NCER-PD datasets are not publicly available, as they are linked to the Luxembourg Parkinson’s Study and its internal regulations. The NCER-PD Consortium is willing to share its available data. Its access policy was devised based on the study ethics documents, including the informed consent form, as approved by the national ethics committee. Requests to access datasets should be directed to the Data and Sample Access Committee via email: request.ncer-pd@uni.lu.
